# Cytoplasmic genome contributions to domestication and improvement of modern maize

**DOI:** 10.1186/s12915-024-01859-4

**Published:** 2024-03-13

**Authors:** Shuai Cao, Huanhuan Zhang, Yang Liu, Yi Sun, Z. Jeffrey Chen

**Affiliations:** 1 State Key Laboratory of Crop Genetics and Germplasm Enhancement, Nanjing Agricultural University, 1 Weigang Road, Nanjing, 210095 China; 2 Shanxi Key Laboratory of Minor Crops Germplasm Innovation and Molecular Breeding, Shanxi Agricultural University, Shanxi, Taiyuan, 030031 China; 3 Department of Molecular Biosciences, The University of Texas at Austin, Austin, TX 78712 USA; 4 Temasek Life Sciences Laboratory, 1 Research Link, National University of Singapore, Singapore, 117604 Singapore

**Keywords:** Maize, Hybrid, Domestication, Improvement, Cytoplasmic genomes, Convergent evolution

## Abstract

**Background:**

Studies on maize evolution and domestication are largely limited to the nuclear genomes, and the contribution of cytoplasmic genomes to selection and domestication of modern maize remains elusive. Maize cytoplasmic genomes have been classified into fertile (NA and NB) and cytoplasmic-nuclear male-sterility (CMS-S, CMS-C, and CMS-T) groups, but their contributions to modern maize breeding have not been systematically investigated.

**Results:**

Here we report co-selection and convergent evolution between nuclear and cytoplasmic genomes by analyzing whole genome sequencing data of 630 maize accessions modern maize and its relatives, including 24 fully assembled mitochondrial and chloroplast genomes. We show that the NB cytotype is associated with the expansion of modern maize to North America, gradually replaces the fertile NA cytotype probably through unequal division, and predominates in over 90% of modern elite inbred lines. The mode of cytoplasmic evolution is increased nucleotypic diversity among the genes involved in photosynthesis and energy metabolism, which are driven by selection and domestication. Furthermore, genome-wide association study reveals correlation of cytoplasmic nucleotypic variation with key agronomic and reproductive traits accompanied with the diversification of the nuclear genomes.

**Conclusions:**

Our results indicate convergent evolution between cytoplasmic and nuclear genomes during maize domestication and breeding. These new insights into the important roles of mitochondrial and chloroplast genomes in maize domestication and improvement should help select elite inbred lines to improve yield stability and crop resilience of maize hybrids.

**Supplementary Information:**

The online version contains supplementary material available at 10.1186/s12915-024-01859-4.

## Background

Corn or maize (*Zea mays* ssp. *mays*) is the highest yielding crop, accounting for over 30% of cereal production worldwide [1]. Besides serving as a source of food, feed, and fuel, maize is an experimental model for studying plant evolution and crop domestication [2, 3]. Maize was domesticated from the wild ancestor teosinte (*Z. mays* ssp. *parviglumis*) approximately 9000 years ago near the Balsas river [4] through dramatic changes in morphology and agronomic traits, which transform from long branches, multiple small ears, and fruitcase over grain in teosinte to very few branches with a single ear on each branch and many grains with exposed fruitcase in modern maize [5, 6].

This domestication process may involve one [7] or two teosintes [8] through introgression of several regulatory genes [9]. *teosinte branched1* (*tb1*) encodes a transcription factor of TEOSINTE BRANCHED1/CYCLOIDEA/PROLIFERATING CELL NUCLEAR ANTIGEN FACTOR (TCP) family, whose expression is regulated by an upstream transposable element (TE) in modern maize [10], and can inhibit the outgrowth of axillary buds to control apical dominance [11]. *Teosinte glume architecture1* (*tga1*), encoding a squamosa-promoter binding protein (SBP) transcription factor, is another maize domestication gene. *tga1* affects the binding activities of its protein TGA1, leading to the shift from kernels encased in a hardened fruitcase in teosinte to naked kernels exposed on the ear in modern maize [12]. Domestication can also enhance maize adaptation to higher latitudes through the alteration of some genes involved in photoperiodic flowering, such as *ZmCCT9*, encoding a CCT (CONSTANS, CO-like, and TOC1) domain transcription factor, which regulates photoperiodic sensitivity and flowering [13, 14], and *ZEA CENTRORADIALIS8* (*ZCN8*), the maize florigen gene, which mediates flowering and adaption [15]. In addition to gain or enhanced traits of adaptation and seed quality, some agronomic traits are decreased or lost during domestication. For example, *TEOSINTE HIGH PROTEIN9* (*THP9*) encodes an asparagine synthetase 4 enzyme that is highly expressed in teosinte, but not in the B73 inbred line. The low expression of *THP9* is associated with a deletion of the tenth intron and decreased seed protein content in modern maize [16].

Maize is a genetically diverse crop with a rapidly evolving nuclear genome, probably due to an abundance of transposable elements (TEs) [17, 18] and intensive domestication and breeding for adaptation to a wide range of environments [19, 20]. Re-sequencing analyses of the maize nuclear genome [21–24] have confirmed the origin of modern maize from teosinte and revealed strong selection during domestication and improvement. The application of modern breeding technology has further improved modern maize lines, including several generations and geographical locations of inbred lines used in commercial hybrid production [13, 25, 26].

However, the impact of cytoplasmic genomes on maize evolution and domestication is poorly understood [27–29]. Cytoplasmic genomes are important to growth and development of plants and animals. Mitochondria and chloroplasts are semi-autonomous organelles with their own genomes, providing cell metabolism and photosynthesis in flowering plants. Mitochondrial and chloroplast genomes, also known as mitogenomes and plastomes, respectively, are commonly used as molecular markers for evolutionary studies because their rate of sequence changes is rather constant [30], although the evolution rate of mitochondrial genomes can be 10 times faster than the nuclear genome in animals [31]. Moreover, many common diseases including those of paternal origins are associated with mutations in their mitochondrial genomes [32, 33].

Consistent with the mitochondrial role in animal health, mutations in mitogenome can lead to cytoplasmic nuclear male-sterility (CMS) in plants [34], including maize, many of which rapidly evolve through sequence rearrangements and exchanges [35, 36]. In maize, there are three major CMS types: CMS-C, CMS-T, and CMS-S, which are classified according to the fertility by their respective nuclear restorer-of-fertility (*Rf*) genes [37]. The CMS-C type is related to three chimeric mitochondrial open reading frames (orfs) of *atp9-C*, *atp6-C*, and *coxII-C* [38], and its fertility can be fully restored by the nuclear gene *rf4* [39]. CMS-T is caused by a mutation of *T-urf13* through rearrangement of the mitogenome in the Texas cytoplasm and susceptible to Southern corn leaf blight [40], and the nuclear gene *rf2*, encoding a putative aldehyde dehydrogenase, can restore the pollen fertility in the CMS-T lines [41]. The CMS-S is associated with co-transcription of *orf355*/*orf77* due to rearrangements [42], and *rf3* is a dominant fertility restorer gene for CMS-S [43]. In addition to these CMS types, modern maize accessions are distinguished by the male-fertile cytotypes of NA and NB, which are subject to 16 rearrangements in the mitogenome [36]. The fertile NB type is present in most commercial hybrids [36], while the other fertile NA type, originally identified in the A188 inbred line, is found in most cultivated maize lines in North America [44]. Interestingly, sequence analysis of organellar genomes from a ~ 5000-year-old archaeological maize sample is related to the fertile NB cytotype, suggesting that CMS lines (CMS-S, CMS-T, and CMS-C) evolve after the NB cytoplasm [27], which is different from the conclusion of sequencing eight mitochondrial genomes [45]. This may suggest that the role of mitochondrial and possibly chloroplast genomes during maize domestication has yet to be elucidated.

Here, we compared high-quality whole genome sequencing (WGS) data of 630 maize and relative accessions from China and North America. To investigate the cytoplasmic genome evolution, we analyzed WGS data from representative maize populations from North America, including 61 inbred lines, 23 landraces, and 19 maize relatives from diverse ecogeographic regions in the US and Mexico. We further explored the association of cytoplasmic genome variation during domestication and improvement with key agronomic traits and found candidate loci for breeding applications. Collectively, these data provide new insights into the evolutional history of cytoplasmic genomes during maize domestication and will help further improve maize crop yield and resilience.

## Results

### Evolution of mitochondrial genomes driven by domestication and improvement

**Fig. 1:**
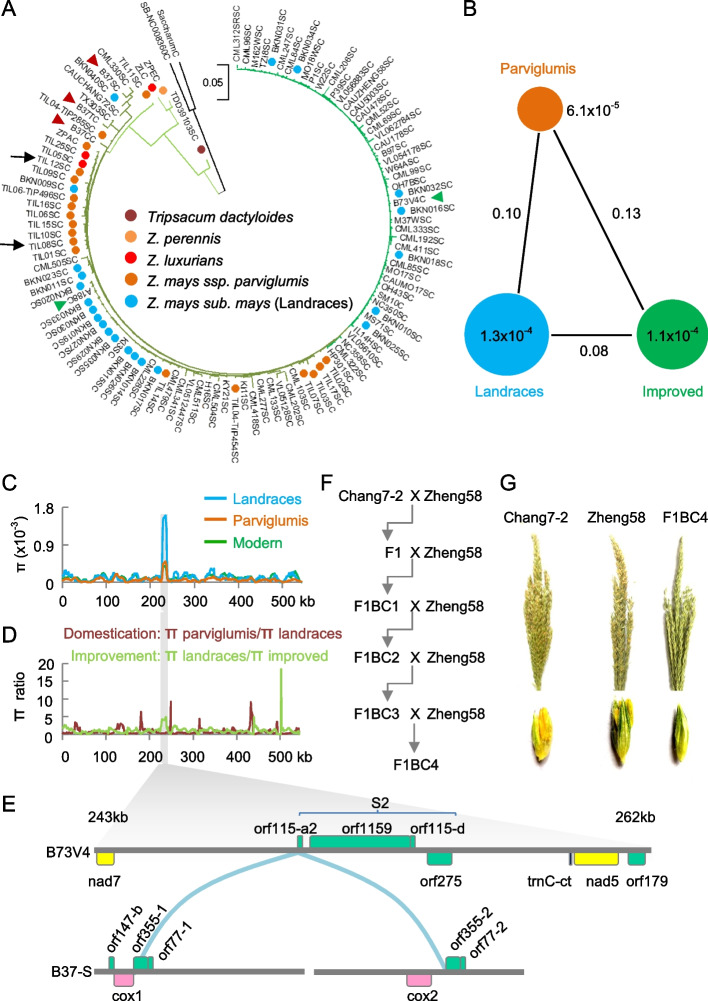
Contribution of the mitogenomes to maize domestication and improvement. **A** Neighbor-joining (NJ) phylogenetic tree reconstructed using SNPs of mitogenomes between different species of *Zea* using *Sorghum bicolor* as an outgroup. Two *Z. parviglumis* accessions (black arrows) were clustered in a clade with CMS cytotypes. Also indicated are representative CMS-S (B37S-KP966116), CMS-T (B37T-KP966117), and CMS-C (B37C-KP966115) (red triangles) cytotypes and fertility NA type (A188-KF241980) and NB type (B73V4) (green triangles) cytotypes. Brach length scale = 0.05. **B** Genetic diversity based on SNPs of mitogenomes among improved lines, landraces, and *Z. parviglumis*. **C** Nucleotide diversity (π) of mitogenomes among improved lines, landraces, and *Z. parviglumis*. **D** Candidate regions of mitogenomes under domestication (brown) and improvement (light green). Dashed line indicates the ratio value of 1. **E** Genomic region of high-nucleotide diversity showing Mt groups of B73V4 (NB) and B37S (CMS-S). **F** Backcross scheme of F1(Chang7-2 × Zheng58) to the recurrent parent Zheng58. **G** Tassel (upper panel) and floret (lower panel) images showing male sterility occurred in the F1BC4

To investigate the variation of mitochondrial (Mt) genomes or mitogenomes during maize domestication and improvement, we analyzed 24 assembled cytoplasmic genomes (Additional 1: Table S1), including 24 fully assembled genomes, from a representative population of *Zea* genus, consisting of 61 maize elite inbred lines, 23 landraces, and 19 wild relatives from diverse eco-geographic regions in the US, Mexico, and China (Additional 1: Table S2). After quality control, 1249 high-quality SNPs of the mitogenomes were identified to construct the neighbor-joining phylogenetic tree (Additional 1: Table S3). Two mitogenomes of sorghum (black branches) were used as an outgroup. The clade next to sorghum consisted of *Tripsacum dactyloides*, *Zea perennis*, *Zea luxurians*, *Zea mays* subsp. *mexicana* (hereafter,* Z*. *mexicana*), *Zea. mays* subsp. *parviglumis* (*Z*. *parviglumis*), and *Zea. mays* subsp. *mays* (*Z*. *mays*) (Fig. 1A); the latter included landraces and improved inbred lines. Notably, two mitogenomes from *mexicana* were clustered in a subgroup of *parviglumis*, which indicates its origin from *parviglumis*. Alternatively, there is possible introgression of the latter into the former through naturally outcrossing or human intervention. Interestingly, ~ 69% (16/23) of landraces and ~ 26% (16/61) improved inbred lines were also clustered in the *parviglumis* subgroup. This result suggests that the mitogenome of *parviglumis* has largely been retained in the landraces but gradually lost among improved inbred lines probably via modern breeding.

Maize mitogenomes can be classified into fertile (NA and NB) and cytoplasmic-nuclear male-sterility (CMS-S, CMS-C, and CMS-T) cytotypes [29]. The mitogenome of an archaeological maize SM10 resembles the NB cytotype [27], indicating that maize NB cytotype appeared prior to the domestication of landraces. SM10 is estimated to have existed ~ 5000 years ago, and landraces began to spread into the temperate zone of the US ~ 4000 years ago [21]. This is supported by the phylogenetic tree (Fig. 1A), as SM10 is close to the older NA cytotype, compared to most of the modern maize. Most landraces, including maize NA cytotype (A188) and CMS, were clustered in the *parviglumis*, whereas most modern inbred lines belonged to the NB cytotype. Moreover, landraces were distributed across all branches but more concentrated in the NA and CMS clusters. These results suggest that landraces were domesticated from *parviglumis* and used for the improvement of modern maize inbred lines [11, 19]. The above conclusion was also supported by the phylogenetic tree constructed from mitochondrial genomes of 175 elite inbred lines in China (Additional 2: Figure S1). The data collectively suggest that the NB cytotype of mitogenomes has been selected during modern maize breeding and spread in the temperate zone of the US [21].

### Nucleotypic evolution in mitogenomes

Among diverse cytotypes, analysis of population fixation index (*F*_*ST*_) showed the lowest *F*_*ST*_ value (0.08) between landraces and improved populations, followed by 0.10 between *parviglumis* and improved population, and 0.13 between *parviglumis* and landraces (Fig. 1B). The data suggest modern breeding has a strong effect on mitogenomes between improved populations and landraces and can shorten the time for population improvement during modern breeding [46]. At the nucleotide variation (π) level, mitogenomes showed greater variation in the landraces (1.3 × 10^−4^) and improved lines (1.1 × 10^−4^) than among *parviglumis* accessions (6.5 × 10^−5^) (Fig. 1B). This suggests accelerated variation of mitogenomes during domestication and breeding. The highest level of nucleotide variation (*π*) was localized in a region (243–262 kb) among three populations, especially in landraces (Fig. 1C). This region, along with another region (near 500 kb) (Fig. 1D), may represent a selection sweep during modern maize improvement.

The selection sweep region consisted of *tmC-ct* and eight orfs, including *nad7*, *orf115-a2*, *orf1159*, *orf115-d*, *orf275*, *nad5*, and *orf179* (Fig. 1E). Four orfs, including *orf115-a2*, *orf1159*, *orf115-d*, and *orf275*, were derived from the S2 linear plasmid, which were uniquely related to CMS-S [36]. *orf115-a2* in the maize NB cytotype was collinear to a region spanning *orf335-1/2* in the fully assembled mitogenome of the CMS-S cytotype [36] (Fig. 1E) and another CMS-S cytotype (Additional 2: Figure S2), which is known as the CMS-S cytotype [42]. Landraces contained all cytoplasmic cytotypes, including *parviglumis*, CMS (CMS-T, CMS-C, and CMS-S), and fertile NA and NB cytotypes. These results suggest the evolution and rearrangement of mitogenomes between CMS-S and fertile NB cytotypes from domestication of landraces to breeding selection of the favorable NB cytotype in modern maize.

For example, a common commercial hybrid ZD958 in China was produced by crossing Zheng58 (a maternal parent) with pollen from Chang7-2 (CMS-S cytotype) [47]. In the reciprocal backcrossing scheme using Chang7-2 as a maternal parent and Zheng58 as a paternal parent (Fig. 1F), male sterility was shown clearly in the F1BC4 progeny (Fig. 1G). The data also suggest that the nuclear fertility restorer (*Rf*) gene in Zheng58 is either absent or non-functional to restore the CMS-S fertility in Chang7-2.

### Evolution of chloroplast genomes during maize domestication and improvement

**Fig. 2:**
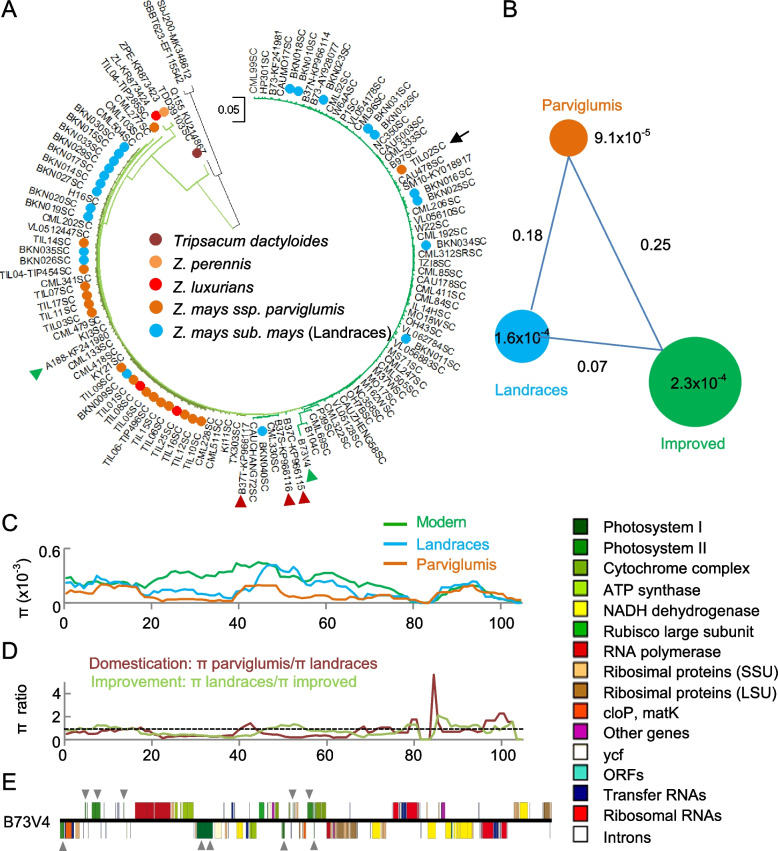
Contribution of plastomes to maize domestication and improvement. **A** Neighbor-joining (NJ) phylogenetic tree reconstructed using SNPs of plastomes between different species of *Zea* using *Sorghum bicolor* as an outgroup. Two *Z. parviglumis* accessions (black arrows) were clustered in a clade with CMS cytotypes. Also indicated are representative CMS-S (B37SC), CMS-T (B37TC), and CMS-C (B37CC) (red triangles) cytotypes and fertility NA type (A188C) and NB type (B73V4C) (green triangles) cytotypes. Brach length scale = 0.05. **B** Genetic diversity based on SNPs of plastomes among improved lines, landraces, and *Z. parviglumis.* **C** Nucleotide diversity (π) of plastomes among improved lines, landraces, and *Z. parviglumis*. **D** Candidate regions of Cp genome under domestication (brown) and improvement (light green). Dashed line indicates the ratio value of 1. **E** Maize plastome of the NB cytotype (Genebank: KP966114) showing the location of photosystem I and II (indicated by triangles) gene clusters (shown on the right with colors)

From chloroplast genomes or plastomes of the same set of genetic materials, we identified 2925 sequence variants, including 2327 SNPs (79.6%) and 598 Indels (20.4%) (Additional 1: Table S4). The phylogenetic neighbor-joining tree revealed that all *parviglumis* and most landraces and maize NA (A188) were clustered in the *parviglumis* clade with one exception (Fig. 2A). One *parviglumis* (accession TIL02) and CMS cytotypes were clustered in another clade, suggesting this *parviglumis* accession may contribute to the CMS phenotype. The same trend was observed among elite inbred lines from China, where improved lines and CMS cytotypes were shown in a separate group (Additional 2: Figure S3). The data indicates that plastomes and mitogenomes are co-selected during maize breeding improvement.

At the population level, landraces and improved populations had the lowest *F*_*ST*_ value (0.07), compared with *parviglumis* and landraces (0.18) and improved populations (0.25), respectively (Fig. 2B). This suggests a close genetic relationship between landraces and improved maize lines, consistent with the conclusion from mitogenomes. The nucleotide variation (π) was the highest in the improved populations (2.3 × 10^−4^) (Fig. 2B), indicating a strong human selection that drives plastome evolution. The nucleotide variation (*π*) in the 20–80-kb region was higher in the landraces than that in *parviglumis* (Fig. 2C and D), while the nucleotide variation in the 20–45-kb region was increased in the improved populations compared with landraces. These regions encode genes and components related to photosynthetic machinery, including photosystem I and II, ATP synthase, rubisco large subunit, ribosomal proteins (SSU and LSU), and ribosomal RNA (Fig. 2E). This result suggests pervasive changes in the chloroplast genome from *parviglumis* in Mexico to increase adaptation to diverse light and temperate environments in the US during modern maize breeding.

### Contribution of cytoplasmic genomes to modern maize breeding

**Fig. 3:**
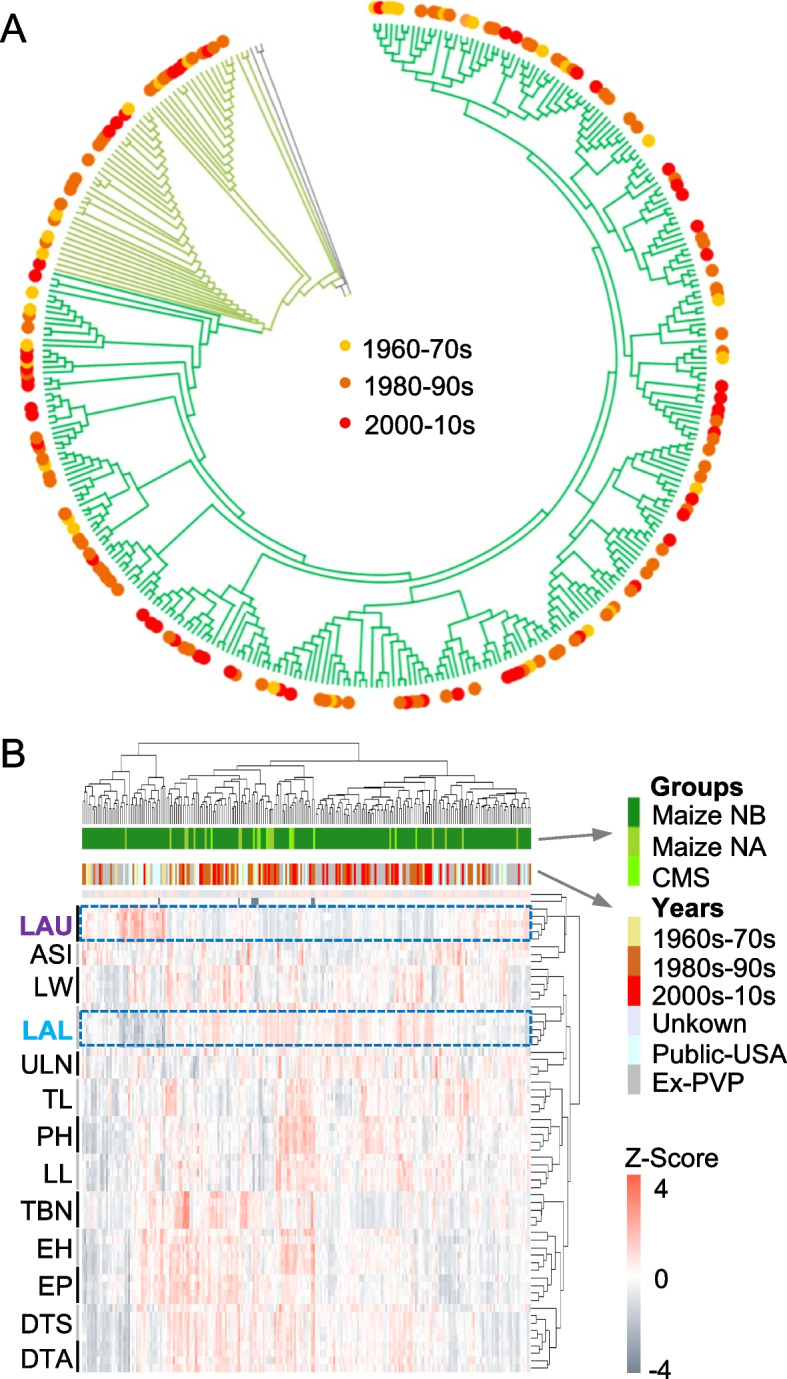
Association of mitogenome diversity with maize breeding lines. **A** Neighbor-joining (NJ) phylogenetic tree reconstructed using SNPs of the mitogenomes between breeding lines in different stages, 1960–1970s (yellow), 1980–1990s (light orange), and 2000–2010s (red). **B** Hierarchical clustering of principal components using 64 agronomic traits among breeding lines in different stages. Abbreviations are LAU (upper leaf angle), ASI (anthesis to silking interval), LW (leaf width), LAL (lower leaf angle), ULN (upper leaf number), TL (tassel length), TBN (tassel branch number), LL (leaf length), PH (plant height), EH (ear height), EP (relative ear position), DTS (days to silking), and DTA (days to anthesis). LAU and LAL are boxed to indicate their antagonistic relationship

Modern breeding has greatly improved corn yield, especially through successful utilization of single-cross hybrids [48, 49]. Although abundant variation of nuclear genomes has been associated with selection during modern breeding, it cannot fully explain many domestication traits involved in energy production, storage, and yield [46]. To explore a role of cytoplasmic genome in modern breeding, we performed a comprehensive analysis of cytoplasmic genomes using a chronological sampling of 343 elite inbred lines (improved populations) from China and North America. The analysis recorded a total of 11,102 variants including 5668 SNPs and 5431 Indels in mitogenome and 1879 variants including 338 SNPs and 1541 Indels in plastome. After quality control, 2440 SNPs of the mitogenome were used to classify these inbred lines into two groups, including a maize NB group and another group with maize NA cytotype and CMS cytotypes (Fig. 3A and Additional 1: Table S5). These inbred lines from different breeding stages were distributed over all branches of the phylogenetic tree (Fig. 3A), different from the nuclear genome variation [46].

To explore the ideotype of mitogenome for modern inbreeding, we further analyzed 64 individual traits of 15 agronomic trait groups among different locations and years [46] (Fig. 3B). While most traits from the same agronomic group were clustered together, the relationship between upper leaf angle (LAU) and lower leaf angle (LAL) is antagonistic (Fig. 3B), in agreement with the ideotype of modern breeding for large LAL and small LAU. Similar discordance occurred between one subgroup of LAL and anthesis to silking interval (ASI) (Additional 2: Figure S4) and another subgroup of other agronomic traits. Some other traits such as relative ear position (EP) (Additional 2: Figure S5), days to silking (DTS) (Additional 2: Figure S6), days to anthesis (DTA), tassel branch number (TBN), and ear height (EH) were positively correlated. Notably, NA cytotype and CMS cytotypes of modern inbred lines had large LAL and small LAU (Fig. 3B), suggesting the contribution of mitogenomes to the ideotype of maize inbred lines.

To further dissect which variation of mitogenome is related to these traits, we next performed GWAS for all 64 traits using mixed linear model-based association analysis (GCTA-MLMA) [50]. After removing variants with > 40% missing calls and minor allele frequency (MAF) of < 0.005, we identified 548 high-quality SNPs in the mitogenome and 45 SNPs in the plastome (Additional 1: Table S6). Significance thresholds for trait association were set at *P*-values of 9.124 × 10^−5^ (0.05/548) and 1.086 × 10^−3^ (0.05/45) for mitogenome and plastomes, respectively. We found three traits, including LAL (Additional 2: Figure S7), TBN (Additional 2: Figure S8), and cob color, stem diameter, and kernel color (Additional 2: Figure S9), were associated with mitogenome diversity at a significant level (*P* < 4.386 × 10^−5^), while plastome SNPs were not associated with these traits (Additional 2: Figure S10).

**Fig. 4:**
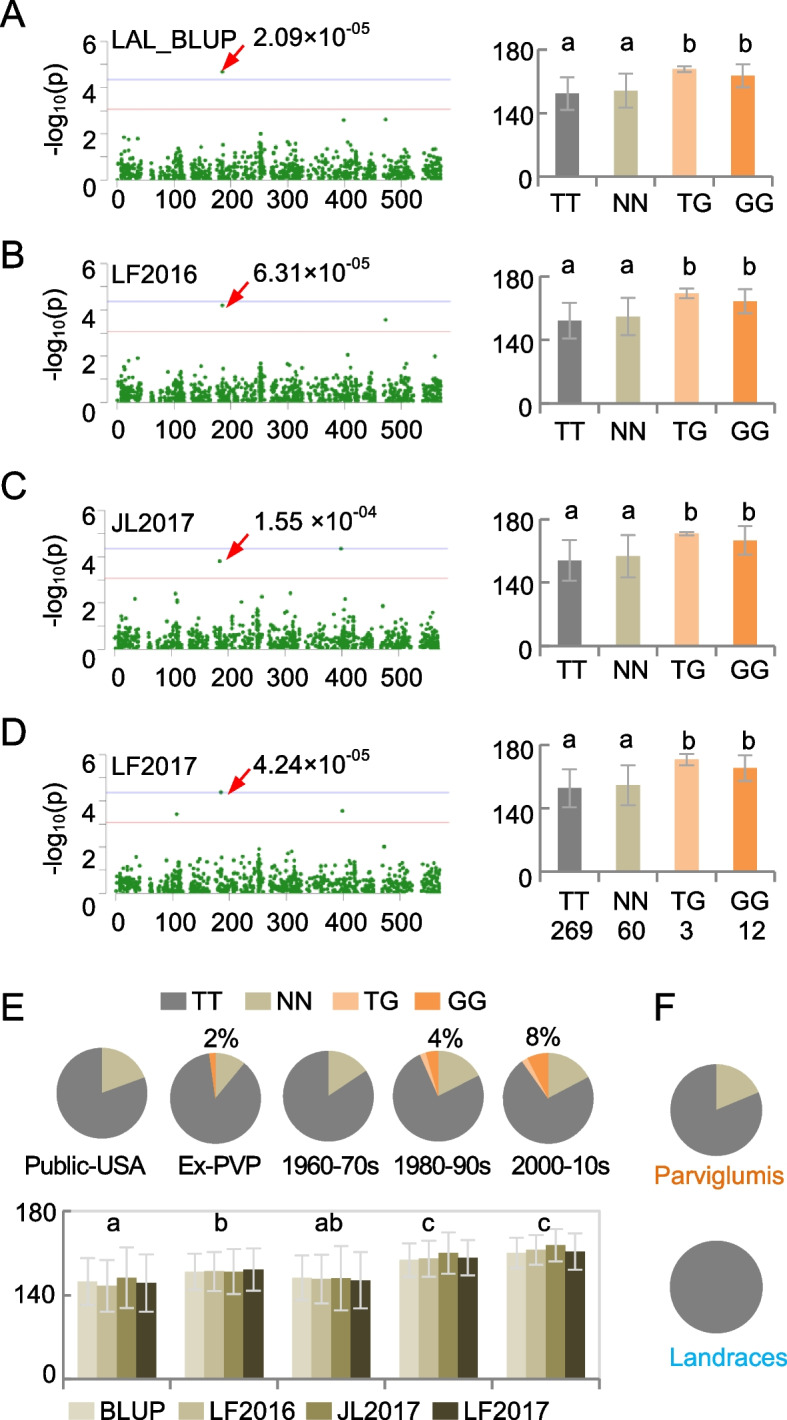
Association of lower leaf angle with mitogenome diversity during maize breeding. **A**–**D** Manhattan plot (left) and mitogenome haplotypes (right) showing association (with a red arrow and *P* value) of lower leaf angle (LAL) of LAL_BLUP (best linear unbiased predictor) (**A**), LAL_LF2016 (**B**), LAL_JL2017 (**C**), and LAL_LF2017 (**D**) with the haplotypes TT, NN, TG, and GG, respectively, in the position of 184,809. The LAL were collected in the city of Langfang in Hebei province in 2016 (LF2016) and 2017 (LF2017), Ledong County in Hainan province in 2016 (HN2016), and Gongzhuling in Jilin province in 2017 (JL2017) in China. The arrow in (**A**) indicates the best linear unbiased predictor value for data of LAL phenotyped across four environments in 2 consecutive years. Different letters indicate statistical significance of *P* < 0.05 (Student’s *t*-test). **E** Proportion of mitogenome haplotypes at 184,809 (upper panel) with the lower leaf angle trait which is one of four listed in **A**–**D** (lower panel) among breeding lines in different stages and regions. Public-USA and Ex-PVP indicate public inbred lines in the US and elite commercial lines with expired Plant Variety Protection Act Certificates mostly released after 2003, respectively. **F** Proportion of mitogenome haplotypes in *Z. parviglumis* (upper panel) and landraces (lower panel)

The mitogenome SNPs located at the position 184,809, is related to genes of *NADH dehydrogenase subunit 1* (*nad1*), *NADH dehydrogenase subunit 2* (*nad2*), and *tRNA-Ala*, and associated with LAL_BLUP (*P* < 2.09 × 10^−05^) (Fig. 4A), LAL_LF2016 (*P* < 6.31 × 10^−05^) (Fig. 4B), LAL_JL2017 (*P* < 1.55 × 10^−04^) (Fig. 4C), and LAL_LF2017 (*P* < 4.24 × 10^−05^) (Fig. 4D). There were four major haplotypes, including TT (269 lines), GG (12 lines), GT (3 lines), and NN (60 lines, *N* = unknown base). The GT and GG haplotypes were related to increased LAL values, compared with haplotypes of TT and NN (Student’s *t*-test, *P* < 0.05). Interestingly, increased frequencies of these haplotypes were coincident with the increased LAL trait during modern maize breeding in both the USA and China (Fig. 4E, Student’s *t*-test, *P* < 0.05).

**Fig. 5:**
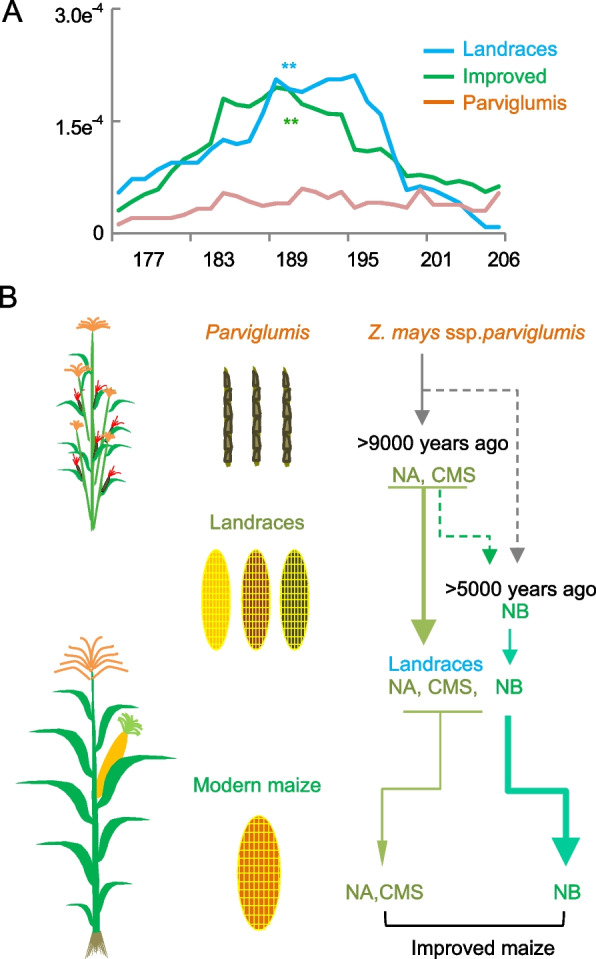
A model for the origin of NA, CMS, and NB cytotypes in maize mitogenomes. **A** Nucleotide diversity (*π*, *y*-axis) of mitogenome (*x*-axis) in the region of 177–206 kb among improved lines, landraces, and *Z. parviglumis*. An asterisk indicates the change between landraces or improved lines and wild maize with statistical significance (*P* < 0.05, two-tailed Student’s *t*-test). **B** Mitogenome of maize was derived from *Z. parviglumis*. Interaction of the *Z. parviglumis* cytoplasm with the nuclear genomes through modern breeding programs produced mitogenome cytotypes including NA and CMS (S, T, and C). Approximately ~ 5000 years ago, NB cytotype of the mitogenome was generated possibly through recombination of NA and CMS (S, T, and C) cytotypes. The combination of NA and CMS cytotypes has been retained through artificial selection, while the NB cytotype became predominant in the commercial inbreed lines associated with maize improvement

However, the GG haplotype was absent in either *parviglumis* or landraces (Fig. 4F), indicating that the GG haplotype could derive from a natural mutation, leading to a larger LAL during modern maize breeding. This notion is supported by the higher level of nucleotide diversity (*π*) in the mitogenome region (177–206 kb) that spans the haplotype in the improved lines than in the landraces and *parviglumis* (Fig. 5A).

### GWAS of 17 agronomic traits from another maize population

Different population may select different loci for shaping traits, and we analyzed another large population of modern maize. To investigate more beneficial alleles for improving breeding, we performed GWAS of cytoplasmic genomes on their trait data [24, 51]. We randomly selected 124 inbred lines with a high depth of re-sequencing data from published data [24] (Additional 2: Figure S11A) and identified a total of 13,420 primary variants from mitogenomes, including 12,024 SNPs (89.6%) and 1396 Indels (10.4%), and a total of 2793 primary variants from the plastomes, including 2194 SNPs (78.6%) and 599 Indels (21.4%). After removing variants with > 40% missing calls and minor allele frequency (MAF) of < 0.005, we identified 924 SNPs in the mitogenome and 144 SNPs in the plastome (Additional 1: Table S7). Based on the SNPs of the mitogenome, the population consisted of two cytoplasmic groups with the NB cytotype being predominant one (Additional 2: Figure S11A). Hierarchical clustering of principal components revealed that the NB cytotype cytoplasm was associated with a group of reproductive traits (vertical box), including HD (heading date), PS (pollen shed), ST (silking time), LNbE (leaf number above the ear), TBN (tassel branch number), and EH (ear height), which is in contrast to the values of these traits in most CMS accessions (horizontal box) (Additional 2: Figure S11B). This result indicates the role of the NB cytoplasm in the development of reproductive traits.

Using genome-wide complex trait analysis-mixed linear model-based association (GCTA-MLMA) [52], we identified the association of GWAS traits with cytoplasmic genomes, using the *p*-values of 5.411 × 10^−5^ (0.05/924) and 8.197 × 10^−4^ (0.05/61) for mitogenomes and plastomes, respectively. The trait of kernel number per row was related to two SNPs at mitogenome positions 109,061 (*P* < 4.14 × 10^−05^) and 109,669 (*P* < 3.03 × 10^−05^), respectively (Additional 2: Figure S11C). Both SNPs are located in the region encoding *nad1*, *nad2*, *tRNA-Ala*, and ribosomal protein S3 (*rps3*), while the kernel width was associated with two other mitogenome loci at 103,594 (*P* < 4.83 × 10^−06^) and 445,983 (*P* < 5.01 × 10^−10^), respectively (Additional 2: Figure S11D). The SNPs located at 103,594 are associated with the *nad1*, *nad2*, *tRNA-Ala* region, and the SNPs located at 445,983 are related to the *nad1* and *nad2* region. In the plastome, the ear leaf width (ELW) was associated with a SNP locus at 52,460 (*P* < 3.44 × 10^−4^), which is located at the ~ 300 bp upstream of *NADH dehydrogenase D3* (*ndhC*) (Additional 2: Figure S11E). These data from two large populations collectively suggest co-evolution between cytoplasmic and nuclear genomes during maize domestication and improvement.

## Discussion

Cytoplasmic genomes (plasmon) of mitogenomes and plastomes with origins of bacterial endosymbionts are essential for life activities of carbon cycle and energy metabolism, including photosynthesis, cellular respiration, and ATP synthesis in flowering plants. In contrast to the nuclear genome, cytoplasmic genomes evolve rapidly through rearrangement to create mutations related to many common diseases of maternal inheritance in mammals [32, 33] and cytoplasmic nuclear male-sterility (CMS) in plants [34].

Maize, the highest-yielding crop, was domesticated from tropical wild ancestor teosinte (*Z. parviglumis*) approximately 9000 years ago near the Balsas river, spreading from the temperate zone to more than the 50th parallel north. This requires wide adaptation to critical limiting factors of light and temperature through adjustment of photosynthesis and energy metabolism during domestication and breeding improvement. We found that 90% or more modern elite inbred lines possess the fertile NB cytotype. Moreover, mitogenomes of *Z. parviglumis* populations were clustered in the subgroups of fertile NA and CMS, suggesting that the fertile NA and CMS cytotypes were generated before landraces of maize were domesticated from *Z. parviglumis* in Mexico [21, 22]. However, the maize NB cytotype has not been found in *Z. parviglumis* populations, which suggests that the NB cytotype has been distinctively produced during modern maize breeding (Fig. 1A). Analysis of ancient DNA from a > 5000-year-old maize (SM10) [27] found the fertile NB cytotype of mitogenome, suggesting that the fertile NB cytotype was formed before the maize was brought to the southwestern USA [26]. These data let us to propose the origin of the NB cytotype between 5000 and 9000 years during early domestication (Fig. 5B). In contrast, CMS and fertile NA cytotypes could be formed before maize domestication along with the *Z. parviglumis* population. According to integration analysis of the above archaeological evidence and genetics, The NB cytotype may be derived directly from Z. *parviglumis* or through possible rearrangement(s) of the NA and CMS (S, T, and C) cytotypes.

The fertile NB cytotype of mitogenomes is popular among maize landraces, which has been selected during maize breeding as a predominant (> 90%) group in modern elite inbred lines and nearly all of commercial hybrids [36]. The discovery and application CMS-T in modern hybrid breeding was a success but did not last long [40, 53], partly because these CMS (T, C, and S) cytotypes such as CMS-T that is highly susceptible to bacterial blight infection [40]. Furthermore, restoration lines for the CMS-C cytotype are uncommon, and the male sterility of CMS-S cytotype is unstable [54]. Our results provide genomic evidence for the origin of three CMS cytotypes from *Z. parviglumis*, but not for selection during domestication of modern maize. The introduction of natural variation among *parviglumis* populations can break this bottleneck of narrow genetic background among CMS cytotypes.

There are some exceptions. For example, the plastome of one *Z. parviglumis* accession (TIL02) was clustered in the maize group, despite its mitogenome being relatively distant from the fertile NB group (Figs. 1A and 2A). This is probably because *Z. parviglumis* and maize are naturally outcrossing during the early stages of domestication [55], such that *L. parviglumis* gene flow has contributed to local adaptation during maize improvement [56]. The plastome inheritance may be retained from unequal division of the cytoplasm, independent of mitogenome inheritance [32]. The unequal division and independent inheritance may also lead to the incongruent evolution between nuclear and cytoplasmic genomes. Notably, the increased nucleotypic diversity of cytoplasmic genomes during maize domestication and improvement is contrary to the evolution features of nuclear genomes (Hufford et al., 2012; Wang et al., 2020). This may indicate the adaptive evolution of mitogenomes in mammals [57], as well as positive or relaxed selection, resulting from increased patterns of nucleotide substitutions as reported in the plastomes of Geraniaceae [58]. The increased nucleotypic variation in the photosynthetic system of the plastome may help adapt rapidly to light and temperature conditions during domestication and improvement (Kistler et al., 2018). Moreover, different cytotypes could have been selected for local adaptation through outcrossing and/or introgression of cytoplasmic genomes by selection and breeding. Similarly, large population of agricultural production and extensive breeding for modern maize could have increased the nucleotide diversity of cytoplasmic genomes compared to nuclear genomes. The association of nucleotypic variation in the mitogenomes and plastomes with different traits in two different populations (Fig. 4 and Additional 2: Figure S10) may also indicate a wide range of effects of the cytoplasmic genomes on photosynthesis, energy metabolism, ideal architecture, and reproductive traits, which play an important role in modern maize breeding and improvement. For example, the association of mitogenomic variation with the lower leaf angles (LAL) suggests the gradual accumulation of this trait through direct or indirect selection during modern maize breeding. These new insights learned from the evolution of cytoplasmic genomes should facilitate and accelerate biological breeding applications in maize production.

## Conclusions

After selection and domestication from one or two tropical wild ancestor teosintes (*Z. parviglumis*), maize cultivation has spread from the temperate zone to more than the 50th parallel north. This wide distribution is accompanied by improved adaptation to critical limiting factors such as light and temperature largely through selection and breeding to enhance photosynthesis and energy metabolism. The genomic basis for maize domestication and breeding improvement depends on convergent evolution between cytoplasmic and nuclear genomes. Using whole genome sequencing data of maize populations and phylogenetic analysis of mitochondrial and chloroplast genomes, we found that the fertile NB cytotype of the mitochondrial genome is associated with the expansion of maize to North America, which gradually replaces the fertile NA cytotype and predominates in more than 90% of modern elite inbred lines. Moreover, population genetics studies have uncovered important roles of mitochondrial and chloroplast genomes during modern maize breeding. Both mitogenomes and plastomes have increased levels of nucleotypic diversity among the genes involved in photosynthesis and energy metabolism, and the cytoplasmic nucleotypic variation in those genes is associated with key agronomic and reproductive traits under diversification and selection of nuclear genomes. These new insights into the evolutional history of cytoplasmic and nuclear genomes during maize domestication and improvement could help select elite inbred lines to improve yield stability and crop resilience of maize hybrids.

## Methods

### Plant materials

Each year, two inbred lines, Chang7-2 and Zheng58, were grown in Taiyuan in summer and Sanya in winter. The Chang7-2 was backcrossed with Zheng58 pollen in summer and winter, respectively. Cytoplasmic nuclear male-sterility lines were identified from backcrossed offspring in each generation.

### Acquisition of WGS data and alignments

We collected a total of 630 whole-genome resequenced accessions of maize and relatives, including 102 accessions from SRA051245 [59], 51 accessions randomly selected from SRA049859 [47], 350 accessions from the PRJNA609577 [46], 124 accessions randomly selected from PRJNA531553 [24], and 3 accessions from PRJNA528290 [55]. The single- or paired-end resequencing reads were downloaded from NCBI Short Reads Archive by the fastq-dump program using the SRA-toolkit (version 2.10.5, https://ftp-trace.ncbi.nlm.nih.gov/sra/sdk/2.10.5/). All reads were filtered into clean data using NGSQCtookit v2.3 (http://www.nipgr.res.in/ngsqctoolkit.html). All cleaned reads were mapped to the cytoplasmic genome sequence of B37NB (KP966114 and AY506529) using BWA (v0.7.15) with four mismatches allowed per read. Only uniquely mapped paired reads were extracted to create new bam files using Perl scripts. Cleaned reads were used for further analysis, after potential PCR duplicates were removed using the “Samtools rmdup” of the Samtools program (version 1.3.1).

### Alignment of assembled cytoplasmic genomes

Assembled cytoplasmic genomes were downloaded from the genebank of NCBI (see Additional 1: Table S1 for details). The sequence of assembled cytoplasmic genomes was split into 200-bp short fragments with steps of 50-bp using Perl scripts. These short fragments are imitated as reads of WGS data to produce fastq files using Perl scripts. The reads were mapped to the cytoplasmic genome sequence of B73 using BWA (v0.7.15) with four mismatches allowed per rea, and only uniquely mapped paired reads were extracted for further analysis.

### Detection of genetic variation

The Genome Analysis Toolkit (GATK, version 3.5.0) and Picard tools (version 2.0.1) were applied to detect sequence variants. “CombineGVCFs” in GATK was applied to merge raw variant calls. The SNPs and Indels were separately classified using “SelectVariants” in GATK. To reduce the variant discovery rate, the SNP calls were filtered according to the following threshold: QD < 10.0 || MQ < 20.0 || FS > 30.0 || SOR > 3.0 || MQRankSum <  − 2.5 || ReadPosRankSum <  − 3.5 || DP < 5. Retained SNPs were used in further analyses.

### Phylogenetic analysis

SNPs from the mitogenomes and plastomes among maize and its relatives were used to construct a neighbor-joining tree using MEGAX with 1000 replicates for bootstrap and confidence analyses.

### Population parameter estimation

The cytoplasmic genome was scanned in 1-kb window size, and population parameters (π and *F*_ST_) were estimated for each window by VCFtools (https://github.com/vcftools/vcftools). Nucleotide diversity (*π*) was measured with the defined parameters “–window-pi 1,000 –window-pi-step 100.” The average 1-kb window size *π* value was estimated as the genetic diversity. For measurement of population differentiation, *F*_ST_ was calculated using the setting “–fst-window-size 1,000 –fst-window-step 100.”

### Genome-wide association analyses (GWAS)

For the population of 343 accessions with 15 traits [46], GWAS was performed using 1140 high-quality SNPs in mitogenomes and 59 high-quality SNPs in plastome genomes, after removing variants with > 40% missing calls and minor allele frequency (MAF) < 0.005. To control spurious associations, genetic relatedness was estimated using “GCTA-GRM” [52]. GWAS was performed in a mixed linear model-based association analysis (MLMA) in the Genome-wide Complex Trait Analysis (GCTA) program, using effective numbers of 548 and 45 independent SNPs for mitogenomes and plastomes, respectively, and their corresponding *P*-values of 9.124 × 10^−5^ (0.05/548) and 1.086 × 10^−3^ (0.05/45). Significant association signals were identified using the thresholds set by GCTA-MLMA [52].

For the population of 73 accessions associated with 17 traits [51], GWAS was performed using a total of 924 high-quality SNPs in mitogenomes and 61 high-quality SNPs in plastomes, after removing variants with > 40% missing calls and minor allele frequency (MAF) < 0.005). Genetic relatedness was estimated using “GCTA-GRM.” GCTA was performed in “MLMA” [52], using an effective number of independent SNPs of 924 and 61 for mitogenomes and plastomes, respectively, and their corresponding *P*-values of 5.411 × 10^−05^ and 8.197 × 10^−04^. Significant association signals were identified using the thresholds set by GCTA-MLMA [52].

### Supplementary Information

**Additional file 1: Table S1.** List of assembled maize cytoplasmic genomes. **Table S2**. Whole genome resequencing of 630 maize accessions. **Table S3**. High-quality (HQ) SNPs of mitogenomes for evolution analysis. **Table S4**. High-quality (HQ) SNPs of plastomes for evolution analysis. **Table S5**. High-quality (HQ) SNPs of mitogenomes for population genetic analysis. **Table S6**. High-quality (HQ) SNPs of mitogenomes and plastomes for GWAS with 64 individual traits of 15 agronomic trait groups. **Table S7**. High-quality (HQ) SNPs of mitogenomes and plastomes for GWAS with 17 agronomic traits.

**Additional file 2: Figure S1.** Neighbor-joining (NJ) phylogenetic tree reconstructed using SNPs of mitogenomes from elite inbred lines of China. **Figure S2.** Multiple sequence alignment (MSA) in the *orf115-a2* region of the mitogenome of NB and CMS-S cytotypes. **Figure S3.** Neighbor-joining (NJ) phylogenetic tree reconstructed using SNPs of plastomes from elite inbred lines of China. **Figure S4.** Manhattan and Q-Q plots showing significant signals for the anthesis-silking interval (ASI) trait using SNPs of mitogenomes. **Figure S5.** Manhattan and Q-Q plots showing significant signals for the relative ear position (EP) trait using SNPs of mitogenomes. **Figure S6.** Manhattan and Q-Q plots showing significant signals for the days to silking (DTS) trait using SNPs of mitogenomes. **Figure S7.** Manhattan and Q-Q plots showing significant signals for the lower leaf angle (LAL) trait using SNPs of mitogenomes. **Figure S8.** Manhattan and Q-Q plots showing significant signals for the tassel branch number (TBN) trait using SNPs of mitogenomes. **Figure S9.** Manhattan and Q-Q plots showing significant signals for the cob color, stem diameter, and kernel color traits using SNPs of mitogenomes. **Figure S10.** Manhattan and Q-Q plots showing significant signals for the relative ear position (EP) using SNPs of plastomes. **Figure S11.** Genome-wide association analysis with 17 agronomic traits.

## Data Availability

All sequencing data are available under NCBI under the BioProject accession numbers SRA051245 [59], SRA049859 [47], PRJNA609577 [46], PRJNA531553 [24], and PRJNA528290 [55] and accession numbers in Additional 1: Table S1.

## Abbreviations

CMS: Cytoplasmic-nuclear male-sterility
TCP: TEOSINTE BRANCHED1/CYCLOIDEA/PROLIFERATING CELL NUCLEAR ANTIGEN FACTOR
tb1: Teosinte branched 1
tga1: Teosinte glume architecture 1
TE: Transposable element
CCT: CONSTANS, CO-like, and TOC1
ZCN8: ZEA CENTRORADIALIS8
THP9: *TEOSINTE HIGH PROTEIN9*
Rf: Restorer-of-fertility
orf: Open reading frame
mt: Mitochondrion or mitochondrial
cp: Chloroplast
SNP: Single nucleotide polymorphism
MAF: Minor allele frequency
GWAS: Genome-wide association study
GCTA-MLMA: Genome-wide complex trait analysis-mixed linear model-based association
WGS: Whole genome sequencing
LAU: Upper leaf angle
LAL: Lower leaf angle
ASI: Anthesis to silking interval
ELW: Ear leaf width
EP: Ear position
DTS: Days to silking
DTA: Days to anthesis
TBN: Tassel branch number
EH: Ear height
