# Supplementary material for: Cytoplasmic genome contributions to domestication and improvement of modern maize
Source: BMC Biol. 2024 Mar 13;22:64. doi: 10.1186/s12915-024-01859-4 (PMC10938767; doi:10.1186/s12915-024-01859-4)
Supplement: Supplementary file 2 — Additional file 2: Figure S1. Neighbor-joining (NJ) phylogenetic tree reconstructed using SNPs of mitogenomes from elite inbred lines of China. Figure S2. Multiple sequence alignment (MSA) in the orf115-a2 region of the mitogenome of NB and CMS-S cytotypes. Figure S3. Neighbor-joining (NJ) phylogenetic tree reconstructed using SNPs of plastomes from elite inbred lines of China. Figure S4. Manhattan and Q-Q plots showing significant signals for the anthesis-silking interval (ASI) trait using SNPs of mitogenomes. Figure S5. Manhattan and Q-Q plots showing significant signals for the relative ear position (EP) trait using SNPs of mitogenomes. Figure S6. Manhattan and Q-Q plots showing significant signals for the days to silking (DTS) trait using SNPs of mitogenomes. Figure S7. Manhattan and Q-Q plots showing significant signals for the lower leaf angle (LAL) trait using SNPs of mitogenomes. Figure S8. Manhattan and Q-Q plots showing significant signals for the tassel branch number (TBN) trait using SNPs of mitogenomes. Figure S9. Manhattan and Q-Q plots showing significant signals for the cob color, stem diameter, and kernel color traits using SNPs of mitogenomes. Figure S10. Manhattan and Q-Q plots showing significant signals for the relative ear position (EP) using SNPs of plastomes. Figure S11. Genome-wide association analysis with 17 agronomic traits. [file 12915_2024_1859_MOESM2_ESM.pdf]

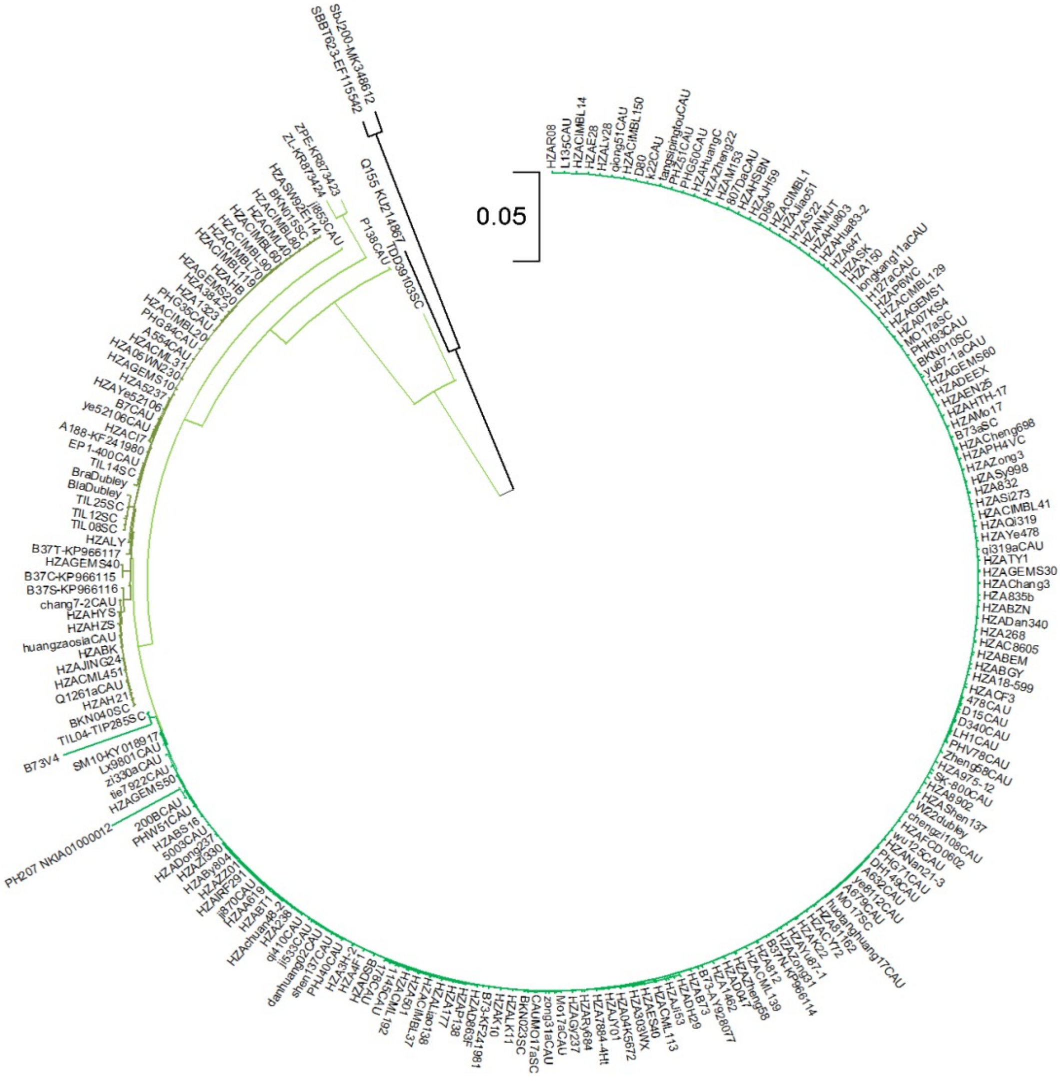

Figure S1. Neighbor-joining (NJ) phylogenetic tree reconstructed using SNPs of mitochondrial (Mt) genomes for elite inbred lines of China. Brach length scale = 0.05.

Sequence	Alignment
**B73V4-NB** |AY506529.1| 250149-250334	AAAGTATACAAGCACATTTCCAATCTACATAAAGATACCA
**CMS-S** |DQ490951.2| 110769-110954	AAAGTATACAAGCACATGTCCAATCTACATAAAGATACCA
**CMS-S** |C_AA055220.1| 110760-110946	AAAGTATACAAGCACATGTCCAATCTACATAAAGATACCA
**CMS-S** |DQ490951.2| 348705-348890	AAAGTATACAAGCACATGTCCAATCTACATAAAGATACCA
**CMS-S** |C_AA055220.1| 348661-348847	AAAGTATACAAGCACATGTCCAATCTACATAAAGATACCA
**Consensus**	aaagtatacaagcacat tccaatctacataaagatacca

Sequence	Alignment
**B73V4-NB** |AY506529.1| 250149-250334	ACCAGGTATCTACTTCAAAGACAGGGCGTCGGCGATCCTC
**CMS-S** |DQ490951.2| 110769-110954	ACCAGGTATCTACTTCAAAGACAGGGCGTCGGCGATCCTC
**CMS-S** |C_AA055220.1| 110760-110946	ACCAGGTATCTACTTCAAAGACAGGGCGTCGGCGATCCTC
**CMS-S** |DQ490951.2| 348705-348890	ACCAGGTATCTACTTCAAAGACAGGGCGTCGGCGATCCTC
**CMS-S** |C_AA055220.1| 348661-348847	ACCAGGTATCTACTTCAAAGACAGGGCGTCGGCGATCCTC
**Consensus**	accaggtatctacttcaaagacagggcgtcggcgatcctc

Sequence	Alignment
**B73V4-NB** |AY506529.1| 250149-250334	TACTATTAAGAGACAGATAACAATGGTGCCGACAGAGATG
**CMS-S** |DQ490951.2| 110769-110954	TACTATTAAGAGACAGATAACAATGGTGCCGACAGAGATG
**CMS-S** |C_AA055220.1| 110760-110946	TACTATTAAGAGACAGATAACAATGGTGCCGACAGAGATG
**CMS-S** |DQ490951.2| 348705-348890	TACTATTAAGAGACAGATAACAATGGTGCCGACAGAGATG
**CMS-S** |C_AA055220.1| 348661-348847	TACTATTAAGAGACAGATAACAATGGTGCCGACAGAGATG
**Consensus**	tactattaagagacagataacaatggtgccgacagagatg

Sequence	Alignment
**B73V4-NB** |AY506529.1| 250149-250334	GACAGAACTGCAGAGAATACCTCTCCGGAGAAGTCCTTAC
**CMS-S** |DQ490951.2| 110769-110954	GACAGAACTGCAGAGAATACCTCTCCGGAGAAGTCCTTAC
**CMS-S** |C_AA055220.1| 110760-110946	GACAGAACTGCAGAGAATACCTCTCCGGAGAAGTCCTTAC
**CMS-S** |DQ490951.2| 348705-348890	GACAGAACTGCAGAGAATACCTCTCCGGAGAAGTCCTTAC
**CMS-S** |C_AA055220.1| 348661-348847	GACAGAACTGCAGAGAATACCTCTCCGGAGAAGTCCTTAC
**Consensus**	gacagaactgcagagaatacctctccggagaagtccttac

Sequence	Alignment
**B73V4-NB** |AY506529.1| 250149-250334	ATGTCTCAAACTAAATAAATCCAAC
**CMS-S** |DQ490951.2| 110769-110954	ATGTCTCAAACTAAATAAATCCAAC
**CMS-S** |C_AA055220.1| 110760-110946	ATGTCTCAAACTAAATAAATCCAAC
**CMS-S** |DQ490951.2| 348705-348890	ATGTCTCAAACTAAATAAATCCAAC
**CMS-S** |C_AA055220.1| 348661-348847	ATGTCTCAAACTAAATAAATCCAAC
**Consensus**	atgtctcaaactaaataaatccaac

Figure S2. Multiple sequence alignment (MSA) in the *orf115-a2* region of the mitogenome of NB and CMS-S cytotypes. E-values ($8e^{-95}$) were estimated by blastn suite-2sequences (https://www.ncbi.nlm.nih.gov/). B73V4-NB mitogenome sequence shown is located at 250,149-250334 adjacent to *orf115-a2*. The second and third sequences are CMS-S mitogenomes of DQ490951.2 and C_AA055220.1, located at 110,769-110,954 and 110,760-110,946 adjacent to *orf355-1*, respectively. The fourth and fifth sequences are CMS-S mitogenomes of DQ490951.2 and C_AA055220.1, located at 348,705-348,890 and 34,8661-34,8847 adjacent to *orf355-1*, respectively. DQ490951.2 and C_AA055220.1 were downloaded from https://www.ncbi.nlm.nih.gov/ and https://ngdc.cncb.ac.cn/, respectively.

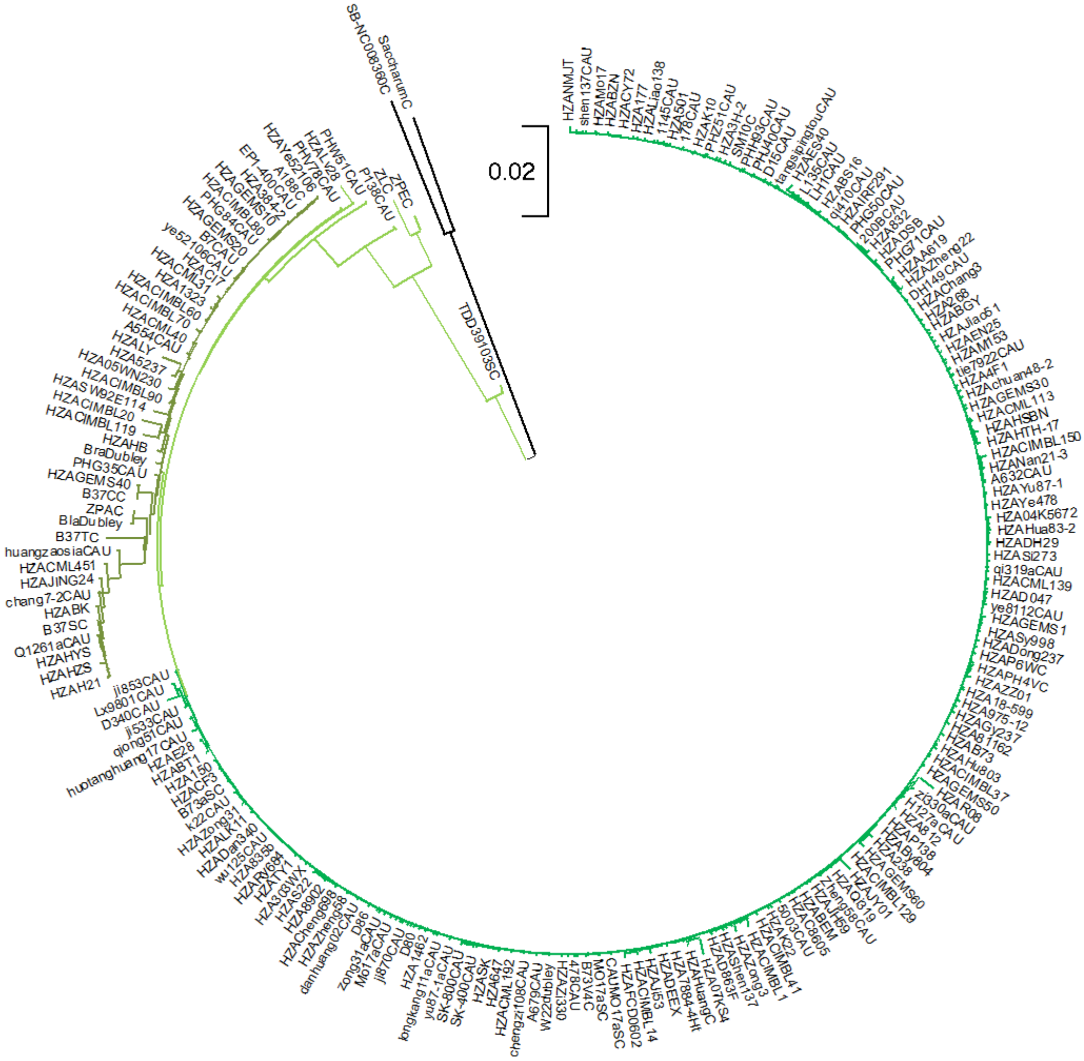

Figure S3. Neighbor-joining (NJ) phylogenetic tree reconstructed using SNPs of chloroplast (Cp) genomes for elite inbred lines of China. Brach length scale = 0.02.

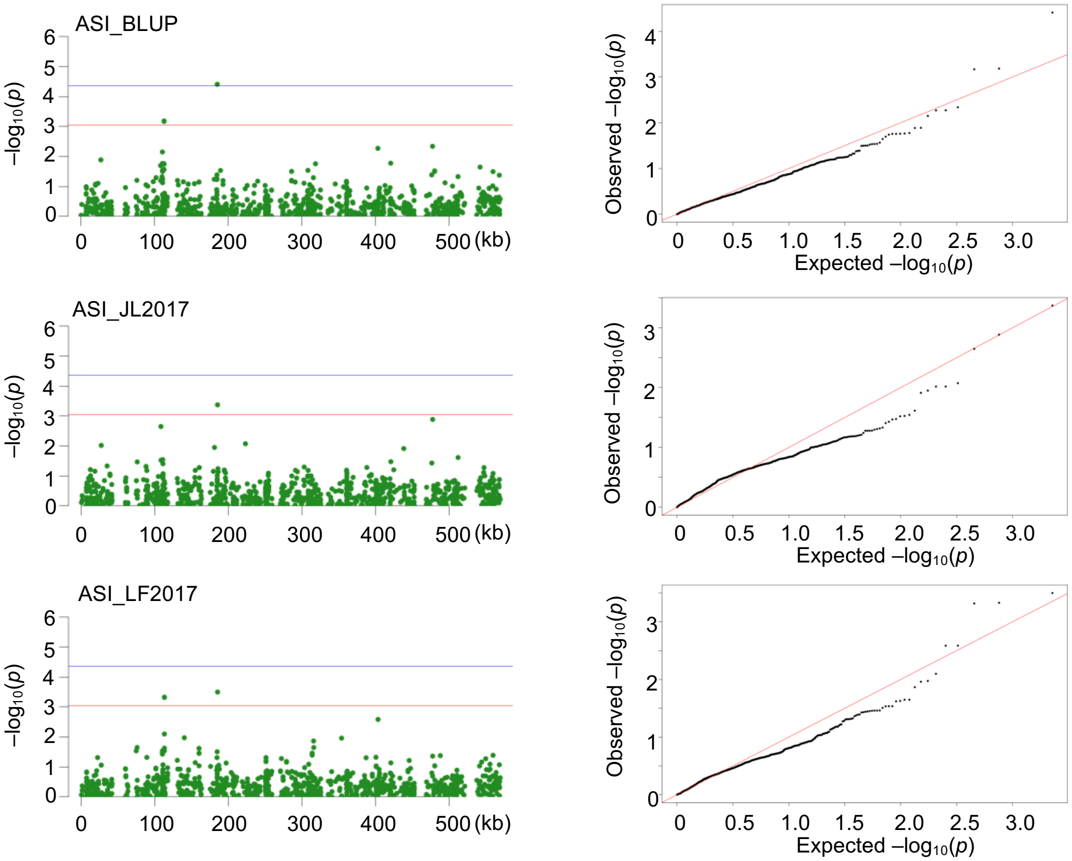

Figure S4. Manhattan and Q-Q Plots for anthesis-silking interval (ASI) based on the SNPs of Mt.

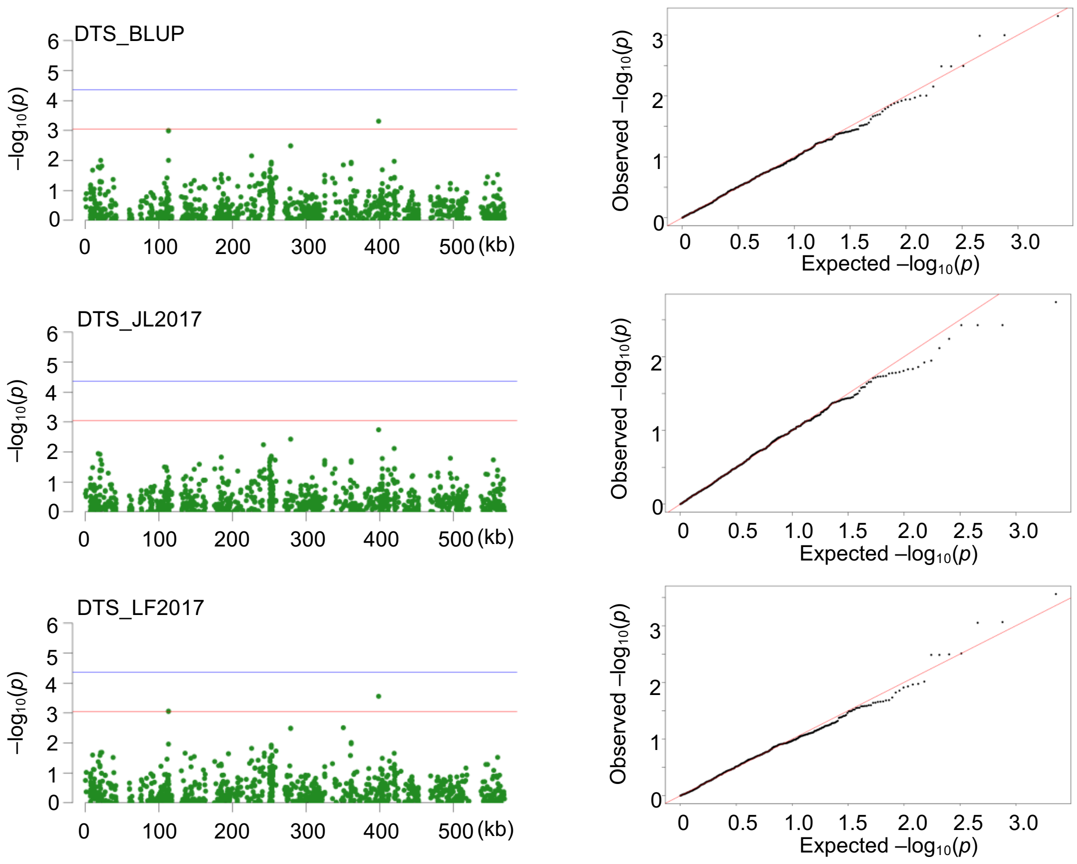

Figure S5. Manhattan and Q-Q Plots for days to silking (DTS) based on the SNPs of Mt.

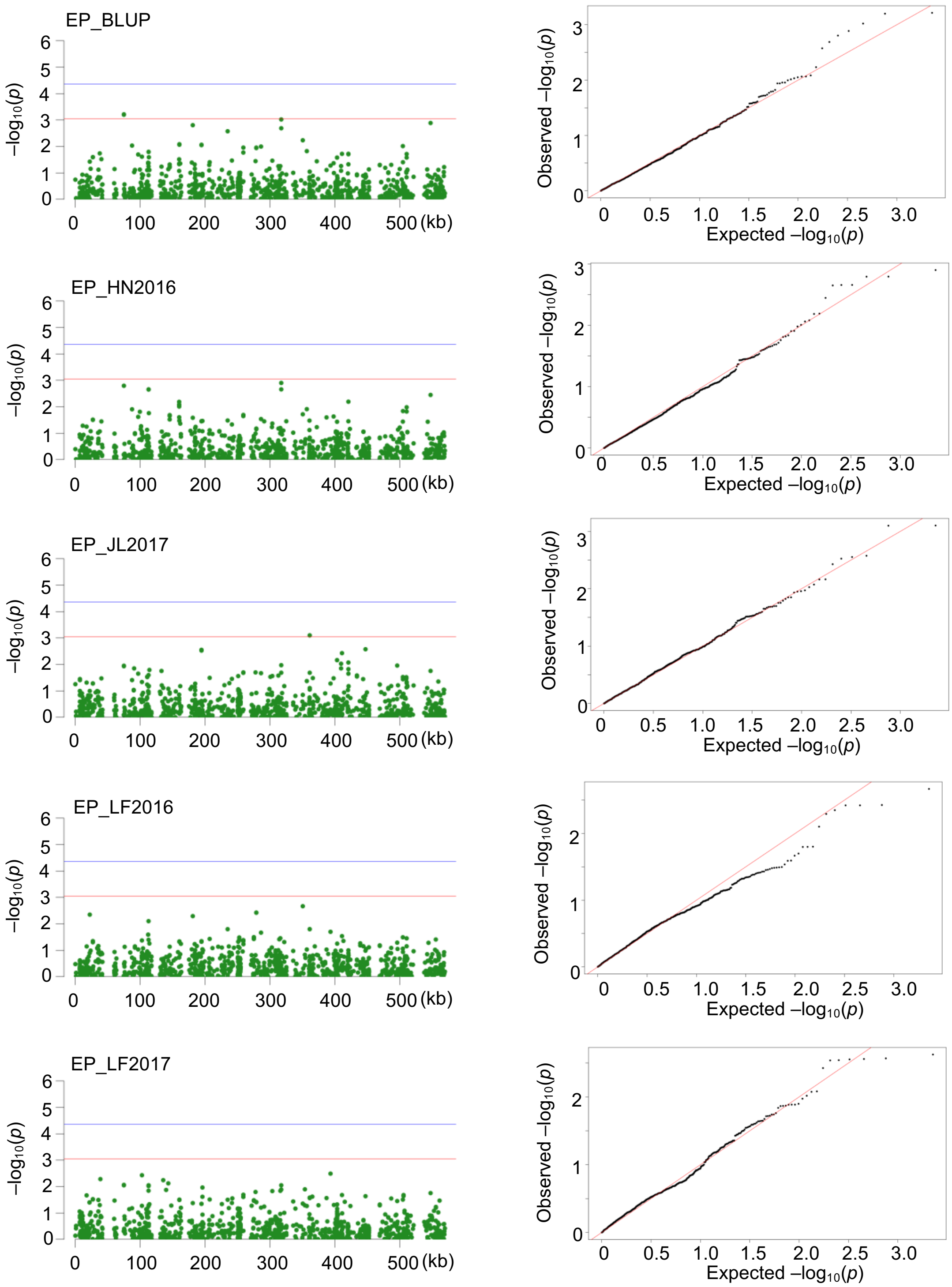

Figure S6. Manhattan and Q-Q Plots for relative height of the ear (EP) based on the SNPs of Mt .

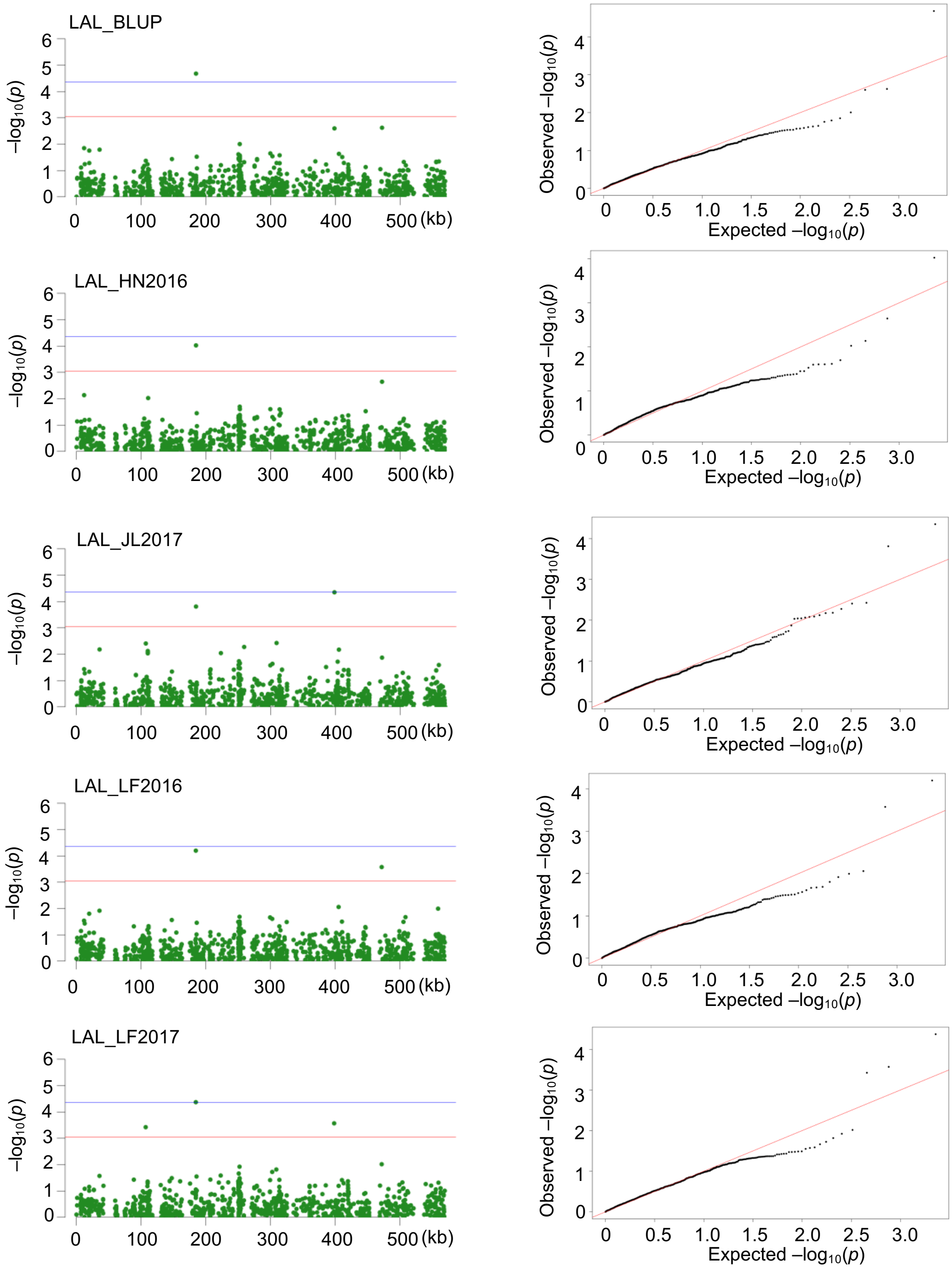

Figure S7. Manhattan and Q-Q Plots for lower leaf angle (LAL) based on the SNPs of Mt.

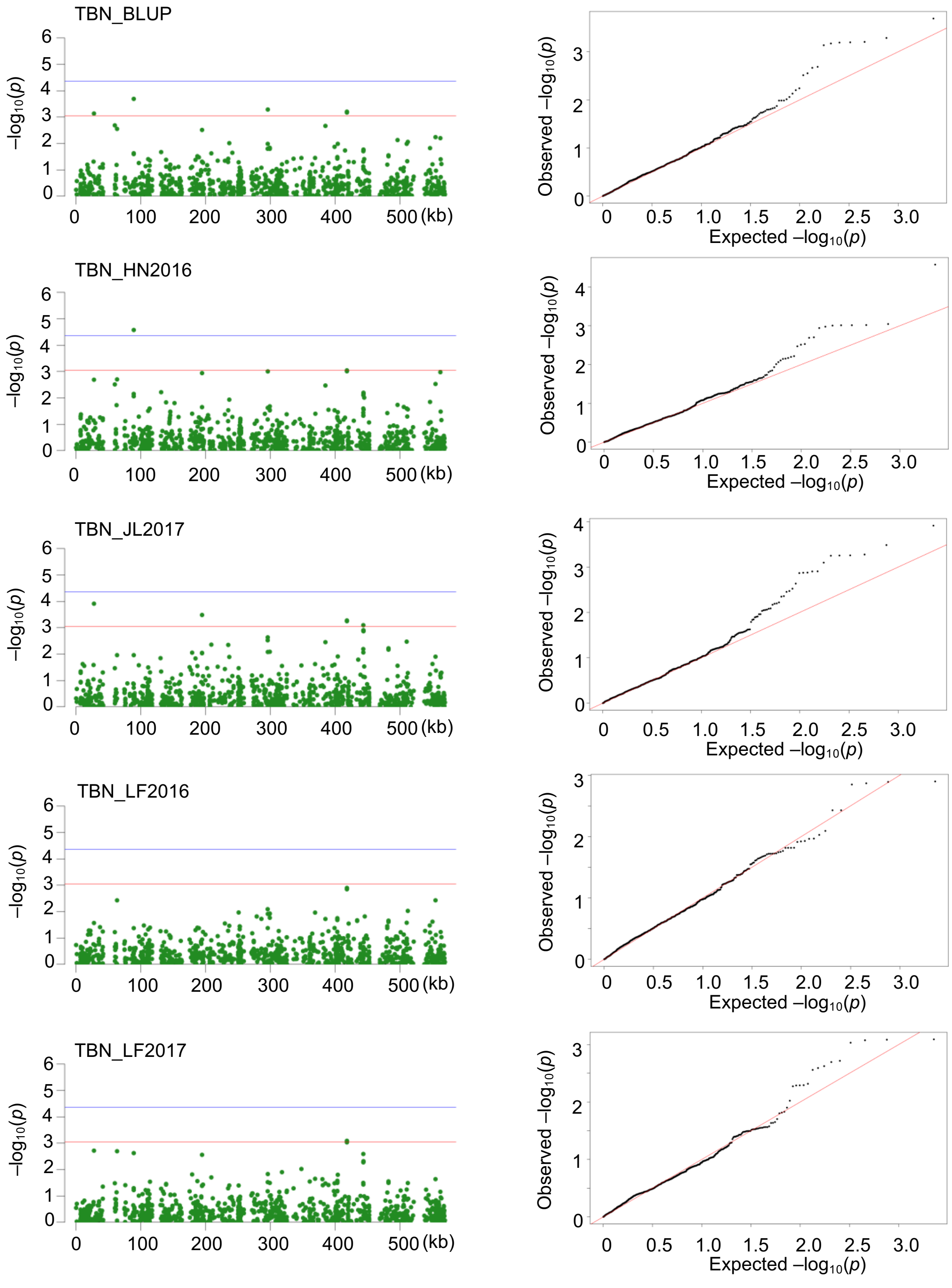

Figure S8. Manhattan and Q-Q Plots for tassel branch number (TBN) based on the SNPs of Mt.

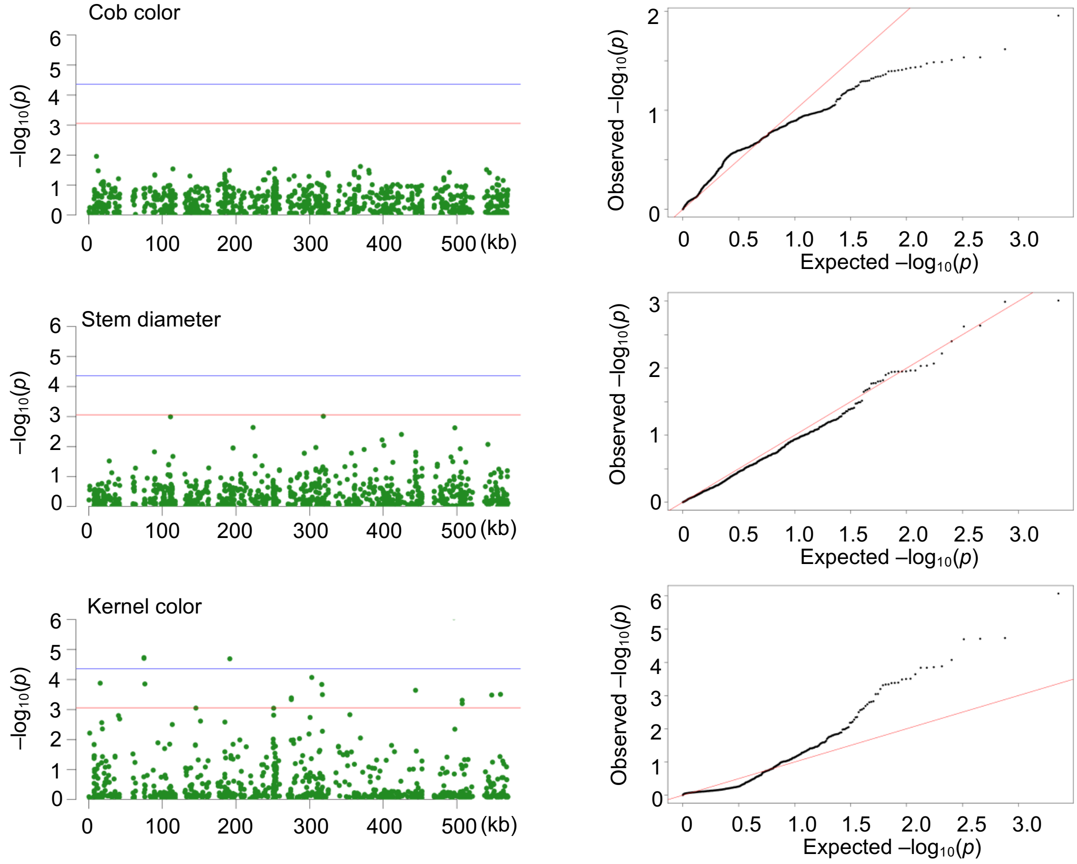

Figure S9. Manhattan and Q-Q Plots for Cob color, Stem diameter, Kernel color based on the SNPs of Mt.

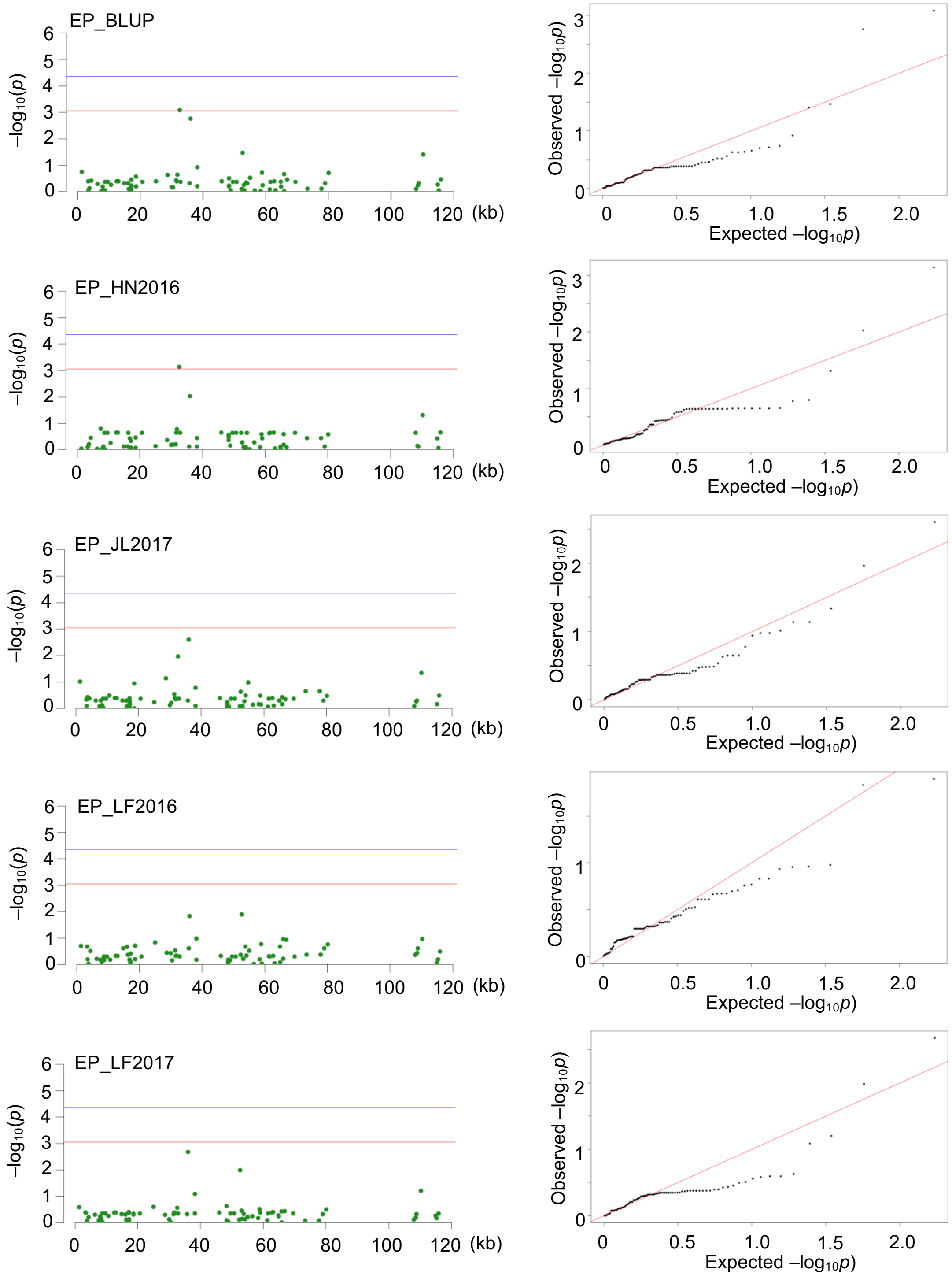

Figure S10. Manhattan and Q-Q Plots for relative height of the ear (EP) based on the SNPs of Cp.

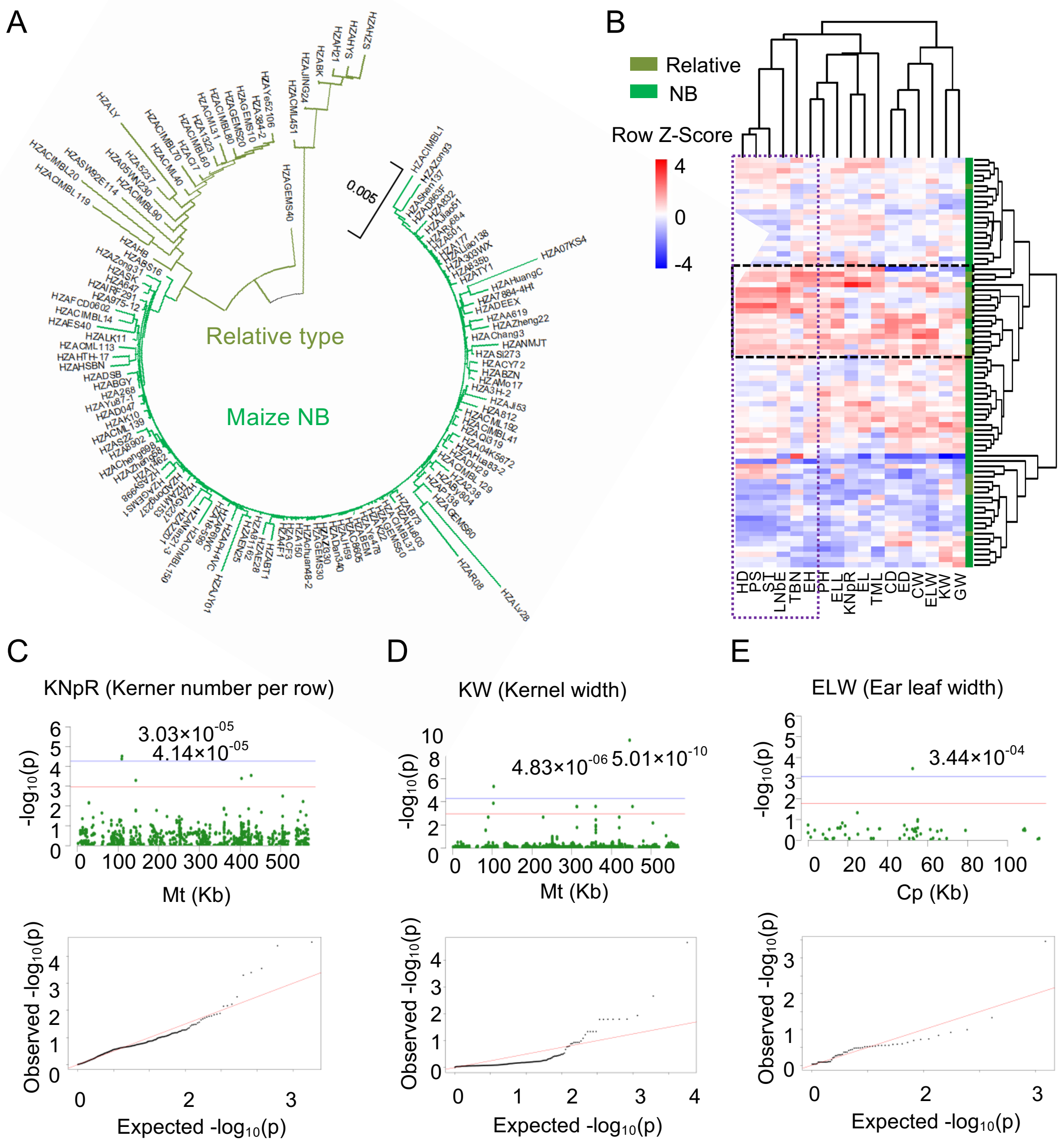

Figure S11. Genome-wide association analysis with 17 agronomic traits.
(A) Neighbor-joining (NJ) phylogenetic tree reconstructed for an improved population using SNPs of Mt and Cp. Brach length scale = 0.005.
(B) Hierarchical clustering on principal components including subpopulations of an improved population based on the 17 agronomic traits, including HD (heading date), PS (pollen shed), ST (silking time), LNbE (leaf number above the ear), TBN (tassel branch number), EH (ear height), PH (plant height), ELL (ear leaf length), KNpR (kernel number per row), EL (ear length), TML (tassel main axis length), ED (ear diameter), CD (cob diameter), CW (cob weight), ELW (ear leaf width), KW (kernel width), and GW (100-grain weight). Horizontal and vertical boxes indicate the CMS accessions and the reproductive traits discussed in the text.
(C) Significant Manhattan (upper) and Q-Q plots (lower) for kernel number per row (KNpR) in the Mt genome.
(D) Significant Manhattan (upper) and Q-Q plots (lower) for kernel width (KW) in the Mt genome.
(E) Significant Manhattan (upper) and Q-Q plots (lower) for ear leaf width (ELW) in the Cp genome.
